# Enhanced electrical properties in sub-10-nm WO_3_ nanoflakes prepared *via* a two-step sol-gel-exfoliation method

**DOI:** 10.1186/1556-276X-9-401

**Published:** 2014-08-18

**Authors:** Serge Zhuiykov, Eugene Kats

**Affiliations:** 1 Materials Science and Engineering Division, CSIRO, 37 Graham Road, Highett, VIC 3190, Australia

**Keywords:** WO_3_, Layered semiconductors, Nanoflake, Sol-gel, Exfoliation

## Abstract

The morphology and electrical properties of orthorhombic β-WO_3_ nanoflakes with thickness of ~7 to 9 nm were investigated at the nanoscale with a combination of scanning electron microscopy (SEM), energy dispersive X-ray spectroscopy (EDX), current sensing force spectroscopy atomic force microscopy (CSFS-AFM, or PeakForce TUNA™), Fourier transform infra-red absorption spectroscopy (FTIR), linear sweep voltammetry (LSV) and Raman spectroscopy techniques. CSFS-AFM analysis established good correlation between the topography of the developed nanostructures and various features of WO_3_ nanoflakes synthesized via a two-step sol-gel-exfoliation method. It was determined that β-WO_3_ nanoflakes annealed at 550°C possess distinguished and exceptional thickness-dependent properties in comparison with the bulk, micro and nanostructured WO_3_ synthesized at alternative temperatures.

## Background

The layered transitional quasi-two-dimensional (Q2D) semiconductor oxides MO_3_ (M = Mo, W), have recently attracted significant interest because they demonstrate quantum confinement effects at the few-layer limit [1,2]. Among them, tungsten trioxide (WO_3_) is an *n*-type semiconductor in an indirect bandgap of 2.6 to 2.9 eV [3] with excellent electrochromic and gasochromic properties [4]. It has electron Hall mobility of ~12 cm^2^V^-1^ s^-1^ at room temperature and responsive to the blue end of the visible spectrum (*λ* < 470 nm) [5]. Extrinsic *n*-doping is therefore not required for WO_3_ to exhibit significant conductivity. Similar to graphene, WO_3_ can be mechanically or chemically exfoliated to provide fundamental layers. However, unlike graphene, which does not have bandgap, Q2D WO_3_ has rather large bandgap, making Q2D WO_3_ nanoflakes more versatile as candidates for thin, flexible devices and potential applications in catalysis [6], optical switches [7] displays and smart windows [8], solar cells [9] optical recording devices [10] and various gas sensors [11]. It has become one of the most investigated functional semiconductor metal oxides impacting many research fields ranging from condensed-matter physics to solid-state chemistry [10].

However, despite great interest of the research and industrial communities to the bulk and microstructured WO_3_, nanoscaled Q2D WO_3_ with thickness less than ~10 nm has received relatively little attention so far compared to its microstructured counterparts and to Q2D transitional metal dichalcogenides MX_2_ (M = Mo, W; X = S, Se, Te). In addition, last year's reports on alternative transitional semiconductor oxide Q2D MoO_3_ have exhibited exceptional thickness-dependent properties and the substantial increased of the charge carriers mobility (up to 1,100 cm^2^ V^-1^ s^-1^) in Q2D MoO_3_[2,12]. It was also recently proven for MoSe_2_ that the reduction of bandgap can be achieved through decreasing the thickness of Q2D nanoflakes down to monolayer [13]. Therefore, realization of WO_3_ in its Q2D form can further engineer the materials' electrical properties, as quantum confinement effects in 2D form will significantly influence charge transport, electronic band structure and electrochemical properties [3]. More importantly, nanostructuring of WO_3_ can enhance the performance of this functional Q2D material revealing unique properties that do not exist in its bulk form [2].

The development of Q2D materials is generally a two-step process, the synthesis of the layered bulk material followed by the exfoliation process [14]. Although there is a wide range of controlled methods of synthesis available to produce different morphologies of WO_3_ nanostructures, such as microwave-assisted hydrothermal [15], vapour-phase deposition [16], sol-gel [17], electron-beam [18] and arc-discharge [19], synthesis of Q2D WO_3_ is a topic that is yet to be widely explored. For instance, in a recent report, it was demonstrated that one possible way of bandgap reduction in bulk WO_3_ is to increase its sintering temperature [20]. However, what is the most favourable sintering temperature for exfoliation Q2D WO_3_ nanoflakes remains largely unexplored.

In this work, we present for the first time new distinguishing thickness-dependent electrical properties of Q2D β-WO_3_ obtained for nanoflakes with thickness below ~10 nm developed via two-step sol-gel-exfoliation method. These properties were mapped without damaging the sample by carefully controlling the sample-tip force. This is performed by using current sensing force spectroscopy atomic force microscopy (CSFS-AFM), also known as PeakForce TUNA™ [21], which allowed simultaneous measurements of the topography and the current flowing between the tip and the sample from the real-time analysis of force-distance curves measured for a tip oscillating in the kilohertz regime, far below the resonance frequency of the cantilever [22]. This technique also provided direct control of the force applied between tip and sample, thus avoiding any damage to the sample or misleading interpretation owing to tip contamination. In addition, new thickness-dependent electrochemical properties of Q2D β-WO_3_ nanoflakes were obtained and compared to the similar properties of the commercially available WO_3_. The electro-catalytic properties of Q2D β-WO_3_ were obtained by investigation samples for hydrogen evolution reaction (HER) from water by linear sweep voltammetry (LSV) and a Tafel plot. The obtained results indicate that Q2D β-WO_3_ nanoflakes are promising electro-catalyst for the HER [6,23,24].

## Methods

Ultra-thin sub-10-nm Q2D WO_3_ nanoflakes were obtained via two-step sol-gel-exfoliation process. All of the following precursors including sodium tungstate dehydrate (Na_2_WO_4_.2H_2_O), hydrogen peroxide (H_2_O_2_, 30%), ethanol, polyethylene glycol (PEG, MW: 20,000), nitric acid (HNO_3_, 65%) and perchloric acid (HClO_4_) were used. Initially, 1 g of Na_2_WO_4_.2H_2_O precursor dissolved in 10 ml de-ionized (DI) water. Then, 6 ml of HNO_3_ was added drop wise to the solution to obtain a greenish yellow precipitation (H_2_WO_4_). After washing with DI water for several times, the remained H_2_WO_4_ was dissolved in 2 ml H_2_O_2_ and stirred at room temperature for 2 h. The procedure was followed by addition of known amount of PEG to obtain a viscous sol and as a result, adherence and homogeneity of the final transparent films can be improved. Then, 30 ml ethanol was added and the sol was stirred for another 2 h. After 1 day of ageing, the prepared sol was deposited on the Au- and Cr-coated Si substrates by using spin-coating instrument (RC8 Spin coater, Karl Suss, Garching, Germany).

The obtained sol-containing thin films were placed in oven at 80°C for a week to achieve the complete gelation. The dried films were subsequently sintered at 550, 650, 700, 750 and 800°C, respectively, for 1 h at the heating rate of 1°C min^-1^. The selection of these temperatures for sintering nanostructured WO_3_ was based on the fact that orthorhombic β-WO_3_ phase can be obtained at various annealing temperatures up to 740°C [20]. Another reason was to investigate at which sintering temperatures mechanical exfoliation is possible and at which particular annealing temperature exfoliation provides the best results. After the samples were sintered and removed from the oven, they were conditioned at room temperature for 7 days. Reproducibility of all sol-gel WO_3_ samples was high. The last phase of the process was to apply mechanical exfoliation in order to obtain extremely thin layers for all further analysis. In the mechanical exfoliation method, scotch-tape was used and the similar procedure applied as per exfoliation of the graphene nanosheets. The reproducibility of exfoliated 2D WO_3_ nanoflakes depended upon the mechanical force applied on the scotch-tape during exfoliation process. Therefore, some of the exfoliated Q2D WO_3_ nanoflakes were thicker than others.

### Structural and physical-chemical characterization

The crystallinity of the sol-gel-developed WO_3_ was characterized by RINT 2100VLR/PC, Rigaku X-ray diffractometer (Shibuya-Ku, Tokyo, Japan) with CuKα radiation (*α* = 0.1542 Å) at angle step of 1° min^-1^. XRD intensities and records were collected using a scintillation detector, and each sample was scanned over the 2-theta range 10° to 80°. Spectral analyses were carried out using Bruker ZRD search match programme, EVA™ (Billerica, MA, USA), and crystalline phases were analysed using the ICDD-JCPDS powder diffraction database. Both the surface morphology and structural configuration of Q2D WO_3_ nanoflakes were evaluated by a Philips XL30 field emission scanning electron microscopy (SEM). Iridium coating was also applied to the sample to improve the quality of the imaging. All the measurements were completed at room temperature. Meanwhile, the local chemical homogeneity of the WO_3_ nanoflakes were conducted by Type N energy dispersive X-ray spectrometer (EDX) (Hitachi Science Systems Ltd., Japan) equipped with JOEL-JSM 5600 LV SEM. Fourier transform infra-red absorption spectroscopy (FTIR) measurements were performed in air at room temperature by using Nicolet 6700 FTIR Spectrometer (Thermo Fisher Scientific, Breda, The Netherlands). Background gas for this examination was N_2_. FTIR spectrometer had the following working parameters during the analysis: IR polarization, zero/no polarization; angle of incidence, 90° perpendicular to the sample; analysing material, KBr and type of detector, MCT detector. During each measurement, the background spectrum was registered and consequently subtracted from the sample spectrum captured to obtain the final spectra. These were studied by employing Omnic Spectroscopy Software Suite. All the spectra were acquired in the following range: 4,000 to 400 cm^-1^. Before experiments, WO_3_ nanostructures were preheated to 200°C for removal of adsorbed moisture and CO_2_ and then cooled down to room temperature. For FTIR measurements, Q2D WO_3_ nanoflakes were also prepared on Au/Si substrate. Ultra-high clean N_2_ was selected as a background gas. It was flowing through the cell containing WO_3_ for 10 min with speed 100 ml min^-1^. After that, WO_3_ nanostructures were exposed to the air for 10 min before any measurements commenced. At each experiment and evaluation, the background spectrum was recorded and subtracted from the sample spectrum obtained [25].

CSFS-AFM measurements were performed to construct and identify the surface profile and simultaneously obtain typical topographical, tunnelling and current/voltage properties of the developed and exfoliated Q2D WO_3_ nanoflakes [26-29]. This procedure was completed using Bruker MultiMode-8 Atomic Force System with installed Peak Force TUNA module (model: MM8-PFTUNA for MultiMode8 AFM system, Rheinstetten, Germany) and the data was analysed by employing NanoScope Analysis software. Raman spectroscopy was used to determine and identify the vibration and rotation information regarding the chemical bonds [30]. μSense-L-532-B Laboratory Raman Analyser (Warsash Scientific Pty Ltd, St, Redfern NSW, Australia) was employed for this purpose. During the testing, CCD detector was cooled down to -60°C. The spectra obtained were studied by RamanReader-M Software (Enwave Optronics Inc, Irvine, CA, USA). Impedance measurements were conducted using a frequency response analyser (AUTOLAB-PGSTAT30, Echo-Chemie, Utrecht, The Netherlands) in the 0.1 M H_2_SO_4_ solution at a room temperature. Lastly, the HER with Q2D WO_3_ nanoflake as the catalyst was measured using standard three-electrode electrochemical configuration in 1.0 M H_2_SO_4_ electrolyte de-aired with Ar, where saturated calomel electrode (Pine Research Instrumentation) and graphite rod (Sigma Aldrich, St. Louis, MO, USA) have been used as reference and counter electrodes, respectively. The reference electrode was calibrated with respect to reversible hydrogen electrode (RHE) using Pt wires as working and counter electrodes. In 1.0 M H_2_SO_4_, E_RHE_ = E_SCE_ + 0.256 V. Potential sweeps were taken with a 5 mV s^-1^ scan rate. Electrodes were cycled at least 30 cycles prior to any measurements.

## Results and discussion

**Figure 1:**
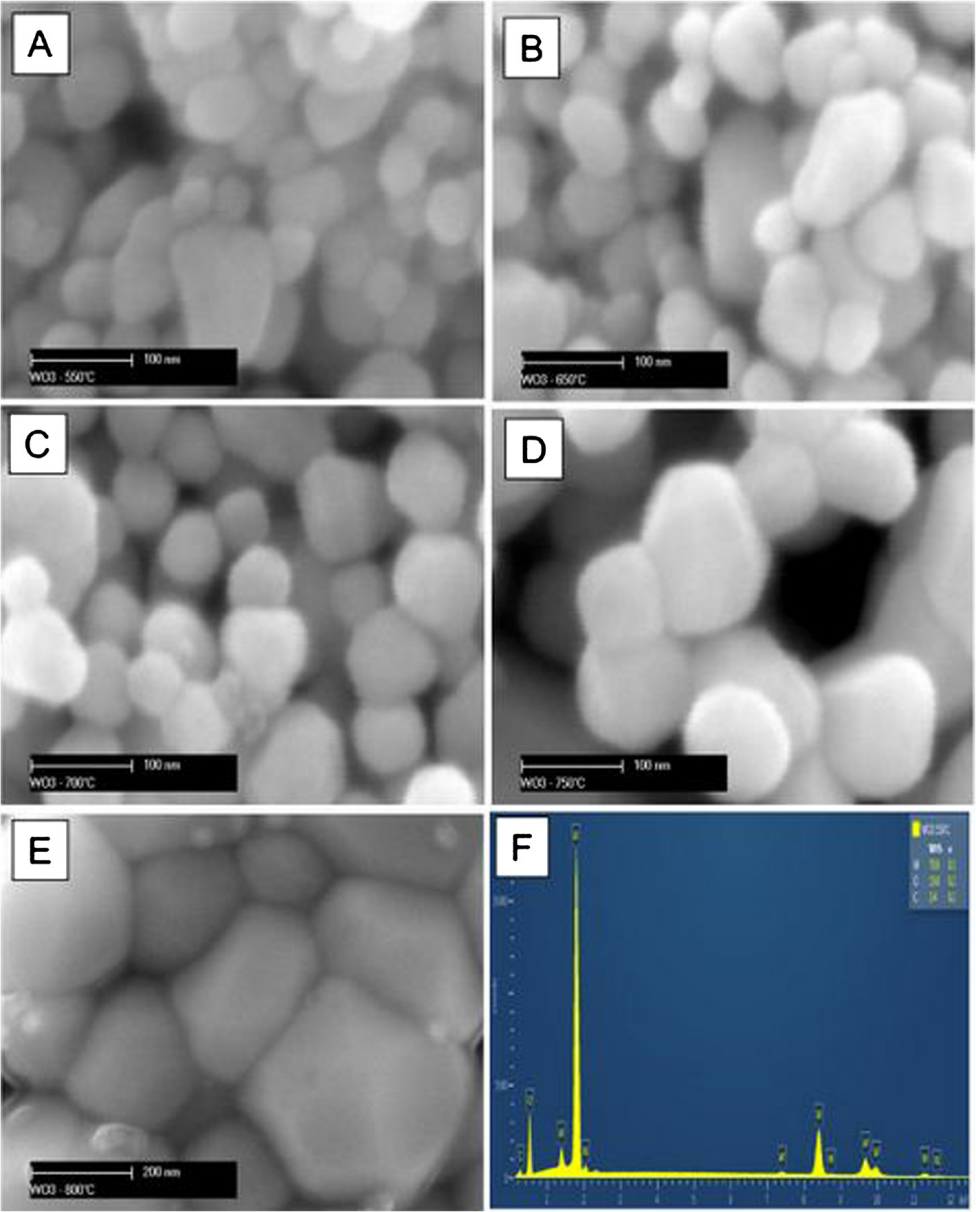
**SEM images of the nanostructured WO**_**3 **_**nanostructures obtained by sol-gel process.** Annealed at 550°C **(A)**, 650°C **(B)**, 700°C **(C)**, 750°C **(D)** and 800°C **(E)**, respectively. EDX analysis for WO_3_ annealed at 550°C **(F)**.

Figure 1 displays SEM images of the sol-gel-developed WO_3_ on Au- and Cr-coated Si substrates, which were sintered at different temperatures. Micrographs of the deposited WO_3_ thin-films revealed the effect of the annealing temperature on the surface morphology. As shown in Figure 1A, the majority of WO_3_ nanoflakes annealed at 550°C were in the range of 20 to 50 nm in length with few larger nanoflakes of ~100 nm. However, as the annealing temperature increased, the morphology of WO_3_ nanoflakes also changed and the average size of the sintered WO_3_ nanoflakes increased (Figure 1B,C,D). For instance, at the sintering temperature of 750°C, the average size of WO_3_ nanoflakes was ~100 to 150 nm. The increase in the sintering temperature seems to have enabled the growth of lager nanoflakes. A further increase in the annealing temperature up to 800°C led to the growth of WO_3_ nanoflakes with average size of ~200 to 400 nm (Figure 1E). This was mainly due to agglomeration of the sintered nanoparticles to form larger crystallites; some of them were larger than 0.5 μm in diameter. The SEM results obtained were in good correlation with independently published results [31]. Subsequent EDX analysis of all the sintered WO_3_ nanostructures confirmed that they comprise a single crystalline phase without impurities. The peaks were narrow with high intensity exhibiting high crystallinity of the developed WO_3_ nanoflakes (Figure 1F). The results of the mechanical exfoliation of all the synthesized WO_3_ nanostructures revealed that it was possible to exfoliate Q2D WO_3_ nanoflakes annealed only at 550° and 650°C, respectively. Higher sintering temperatures ensured the development of strong bonds between adjacent WO_3_ layers preventing exfoliation. Therefore, all other experiments were carried out only on WO_3_ nanoflakes sintered at 550° and 650°C.

**Figure 2:**
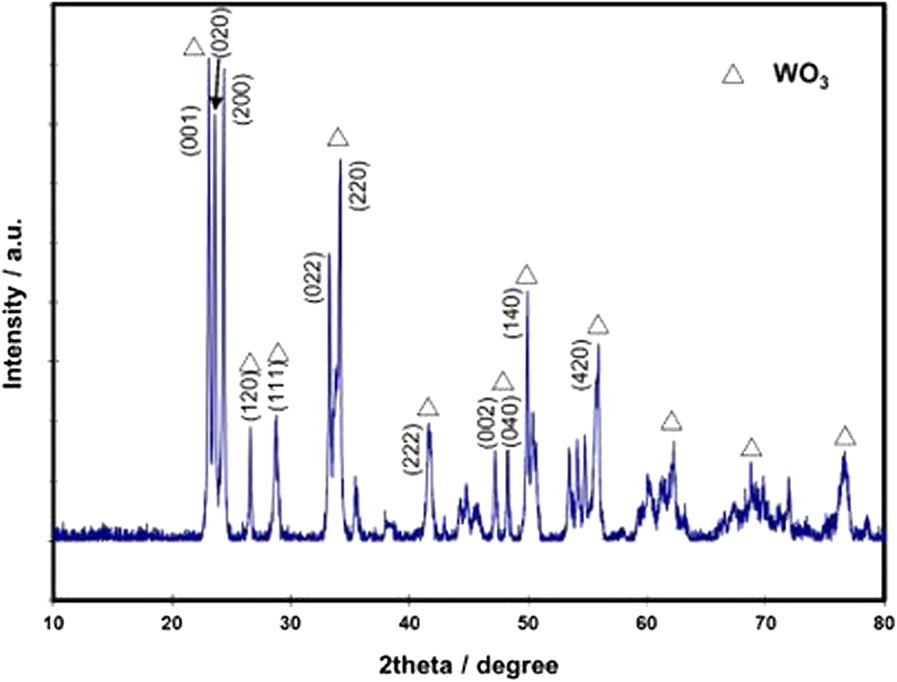
**XRD patterns of the WO**_**3 **_**thin films sintered on Au-covered Si substrate at temperature of 550°C.**

Figure 2 exhibits the XRD patterns for sol-gel prepared WO_3_ nanostructures, which were subsequntly sintered at 550°C. The intense reflection peaks were narrow and sharp indicating that WO_3_ is well crystallized. All reflections were indexed to orthorhombic β-WO_3_ phase (JCPDS card No. 20-1324 with space group *P* and the following lattice parameters: *a* = 7.384 Å, *b* = 7.512 Å, *c* = 3.864 Å). The results obtained were similar to the previously published data for orthorhombic β-WO_3_[3,32,33]. Generally, the orthorhombic phase of WO_3_ is stable in the temperature range of 330 to 740°C [34,35]. No impurities in the developed thin films were detected.

### Characterization of properties of Q2D WO_3_ nanoflakes

Comprehensive information in relation to the developed ultra-thin Q2D WO_3_ and their electrochemical properties, such as chemical structure, oxidation states, adsorption properties etc., must be obtained and optimized in order to achieve their best analytical performance in various applications. For this purpose, CSFS-AFM, FTIR and Raman spectroscopy techniques were used.

**Figure 3:**
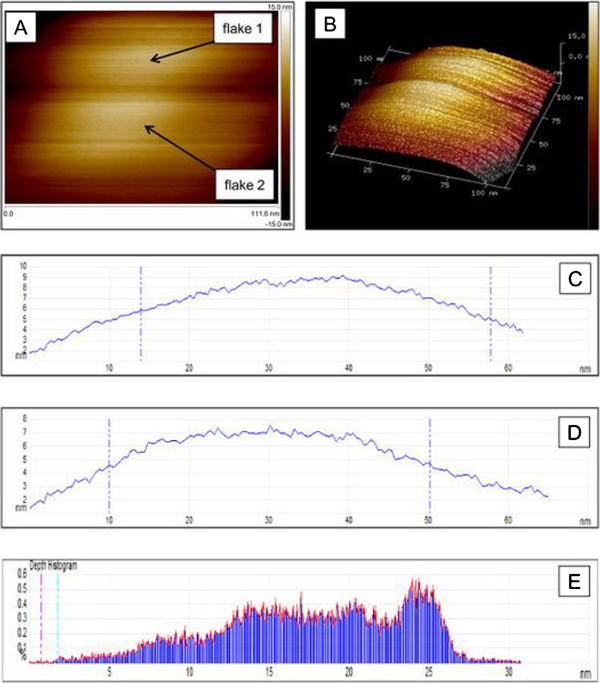
**The topography and morphology of ultra-thin exfoliated Q2D WO**_**3**_**.** AFM images of two exfoliated Q2D WO_3_ nanoflakes (flakes 1 and 2) sintered at 550°C **(A)**, 3D image **(B)**, cross-section height measurements of flake 1 **(C)** and flake 2 **(D)** and depth histogram for flake 2 **(E)**.

The topography and morphology of ultra-thin exfoliated Q2D WO_3_ sintered at 550°C and their characteristics analysed by CSFS-AFM are presented in Figure 3. CSFS-AFM is a relatively new technique for mapping the electrical properties of the developed Q2D nanostructures. Therefore, AFM with Peak Force TUNA™ module was employed to study the topography and morphology of Q2D WO_3_ nanoflakes. Multiple flake morphology of Q2D WO_3_ (Figure 3A) is evidently and consistently observed in all images on the analysing image surface area of 18,365.3 nm^2^. The measured surface area difference was 18.2%. Figure 3B demonstrates 3D image of the general profile and provides information in relation to the structure of two adjacent Q2D WO_3_ flakes with their measured thickness in the range of 7 to 9 nm (Figure 3C,D). It was confirmed that the mechanical exfoliation enables the development of uniformed nanostructure of ultra-thin Q2D WO_3_ nanoflakes with the average determined dimensions of 60 to 80 nm in length and 50- to 60-nm wide. The depth histogram, depicted in Figure 3E, displays the coherency in the structure of the nanoflake. The increased Fowler-Nordheim tunnelling current at the edges between the different nanoflakes represented the dark areas on the image (Figure 3B). This indicates local structural thinning of the oxide during the fabrication, which serves as an insulating area between adjacent active regions. Enhanced current flow is noticeable along the grain boundaries of WO_3_ nanoflake, the peak current with maximum intensity was clearly identified and its measured value was 248 pA. The average tunnelling current was relatively low, corresponding to the changes in WO_3_ nanoflake thickness and small inhomogeneities, as each of the developed Q2D WO_3_ nanoflake consisted of several fundamental layers of WO_3_. Due to the low conductivity of the fabricated Q2D WO_3_ nanoflakes, the adhesion between the PF TUNA tip and the WO_3_ nanoflakes was found to be poor. Noteworthy, the measured thickness of exfoliated Q2D WO_3_ nanoflakes sintered at 650°C was about 15 to 25 nm which is thicker than those exfoliated Q2D WO_3_ nanoflakes sintered at 550°C.

**Figure 4:**
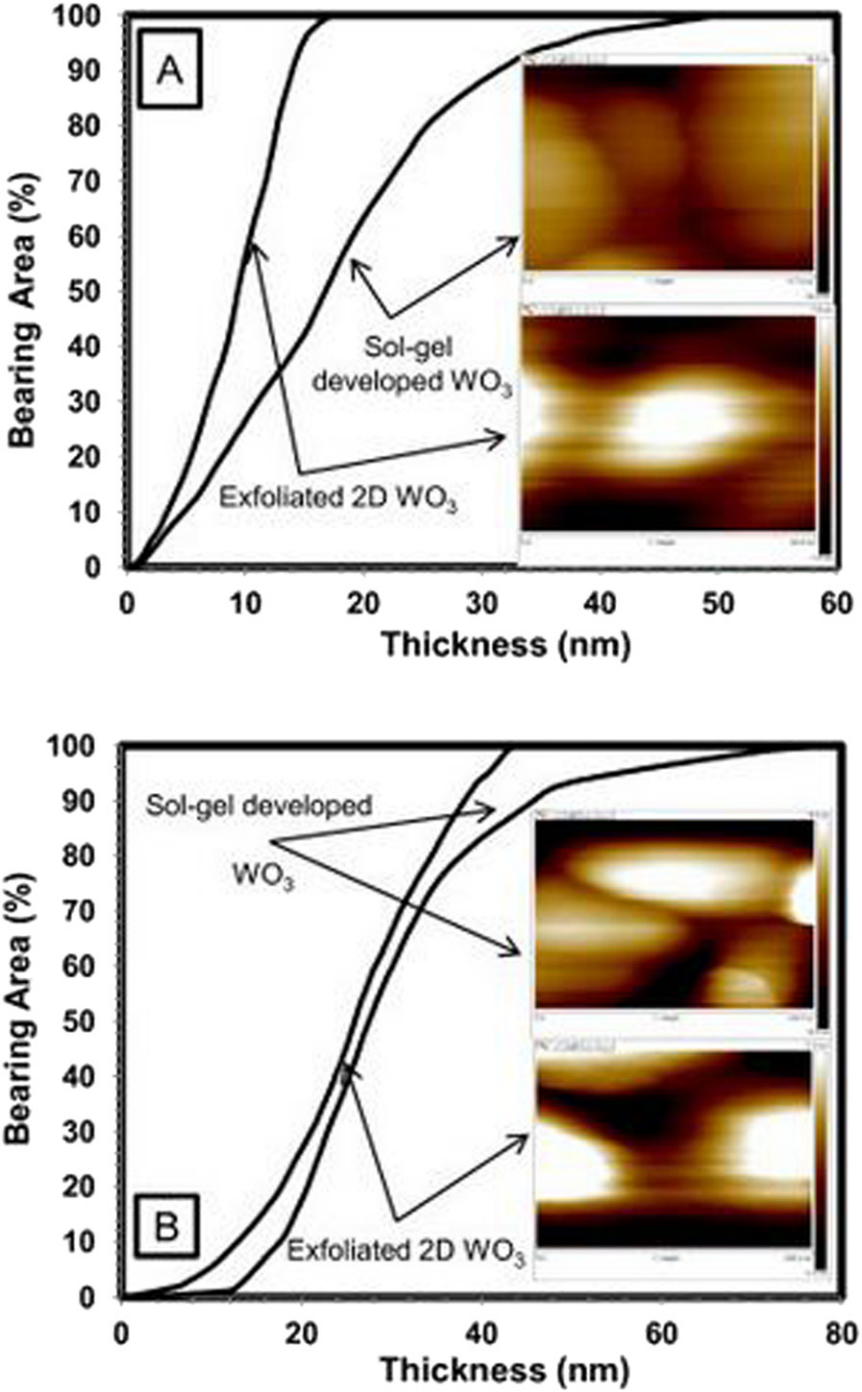
**Bearing area analysis for typical individual sol-gel-developed and exfoliated WO**_**3 **_**nanoflakes sintered at 550°C (A) and 650°C (B).** Inserts: appropriate AFM images of sol-gel-developed and exfoliated WO_3_ nanoflakes, respectively.

It must be taken into account that by using CSFS-AFM, it was possible to analyse not only physical and electrical parameters of the developed Q2D WO_3_ nanostructures with the thickness of less than 10 nm without damaging them, but also mapping measured parameters to the specific morphology of the analysed WO_3_ nanoflakes. Furthermore, the great advantage of this approach can be illustrated by bearing analysis, which represents the relative roughness of a surface in terms of high and low areas. The bearing curve is the integral of the surface height histogram and plots the percentage of the surface above a reference plane as a function of the depth of that below the highest point of the image. Figure 4 elaborates bearing analysis performed on Q2D WO_3_ sintered at 550° and 650°C before and after exfoliation. For the exfoliated Q2D WO_3_ sintered at 550°C (Figure 4A), it is clearly shown that 90% of Q2D WO_3_ nanoflakes had an average particle size of less than 20 nm, whereas prior to exfoliation, 90% of the sub-micron WO_3_ nanostructures comprised flakes with an average particles size of approximately 50 nm. On the other hand, for WO_3_ nanoflakes sintered at 650°C, the average particles size of sol-gel-developed WO_3_ prior to exfoliation was ~75 nm (Figure 4B). Following exfoliation, it was possible to decrease the average particles size down to ~42 nm. Bearing analysis has also confirmed that the exfoliation removes larger nanoagglomerations from the surface of WO_3_ nanostructures and at the same time reduces the thickness of Q2D WO_3_ nanoflakes. These facts suggested that the sintering temperature of 550°C is more suitable than 650°C for mechanical exfoliation and the development of ultra-thin Q2D β-WO_3_ nanoflakes.

**Figure 5:**
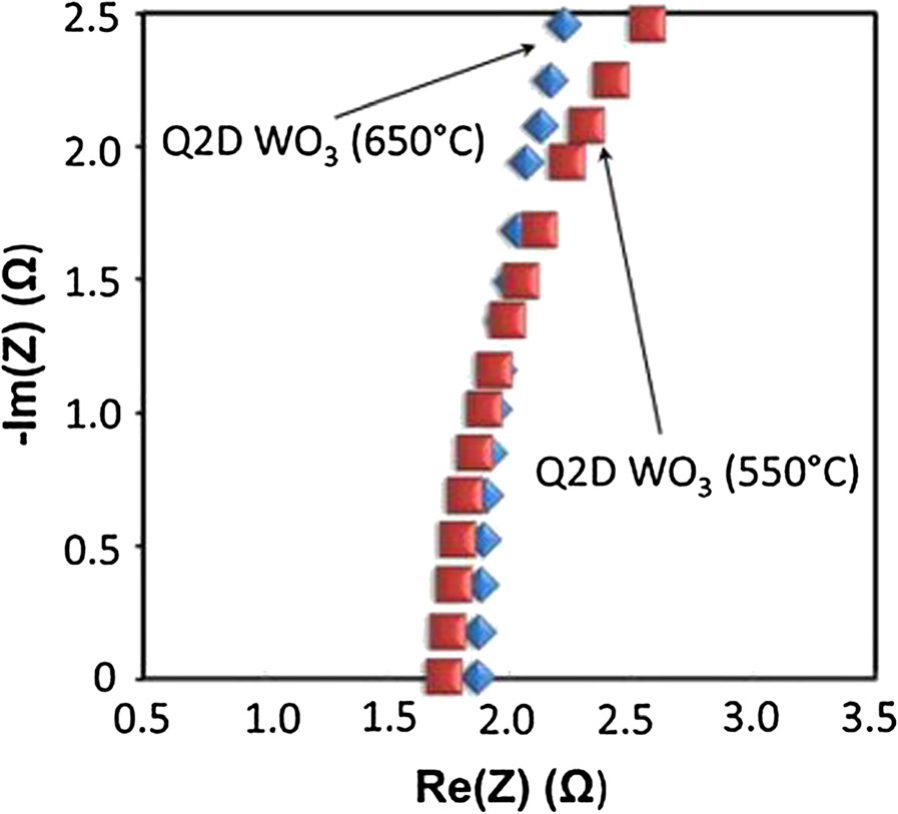
**Nyquist plots of Q2D WO**_**3 **_**nanoflakes annealed at 550°C and 650°C, respectively.**

All impedance measurements were performed on Q2D WO_3_ nanoflakes sintered at 550 and 650°C, respectively. AC impedance measurements were done from 10^6^ to 0.1 Hz with an alternative current of 10 mV and results are presented in Figure 5. We found that there are no significant difference between the impedance recorded for Q2D WO_3_ annealed at 550°C (1.6 ohm) and impedance recorded for Q2D WO_3_ annealed at 650°C (1.8 ohm). Due to very small dimensions, the contribution from the Q2D WO_3_ working electrode into the total impedance confirmed to be very small. The resistance primarily comes from wiring (e.g. cables, alligator clips) and electrolyte, where the resistance of Q2D WO_3_ nanoflakes is negligible.

**Figure 6:**
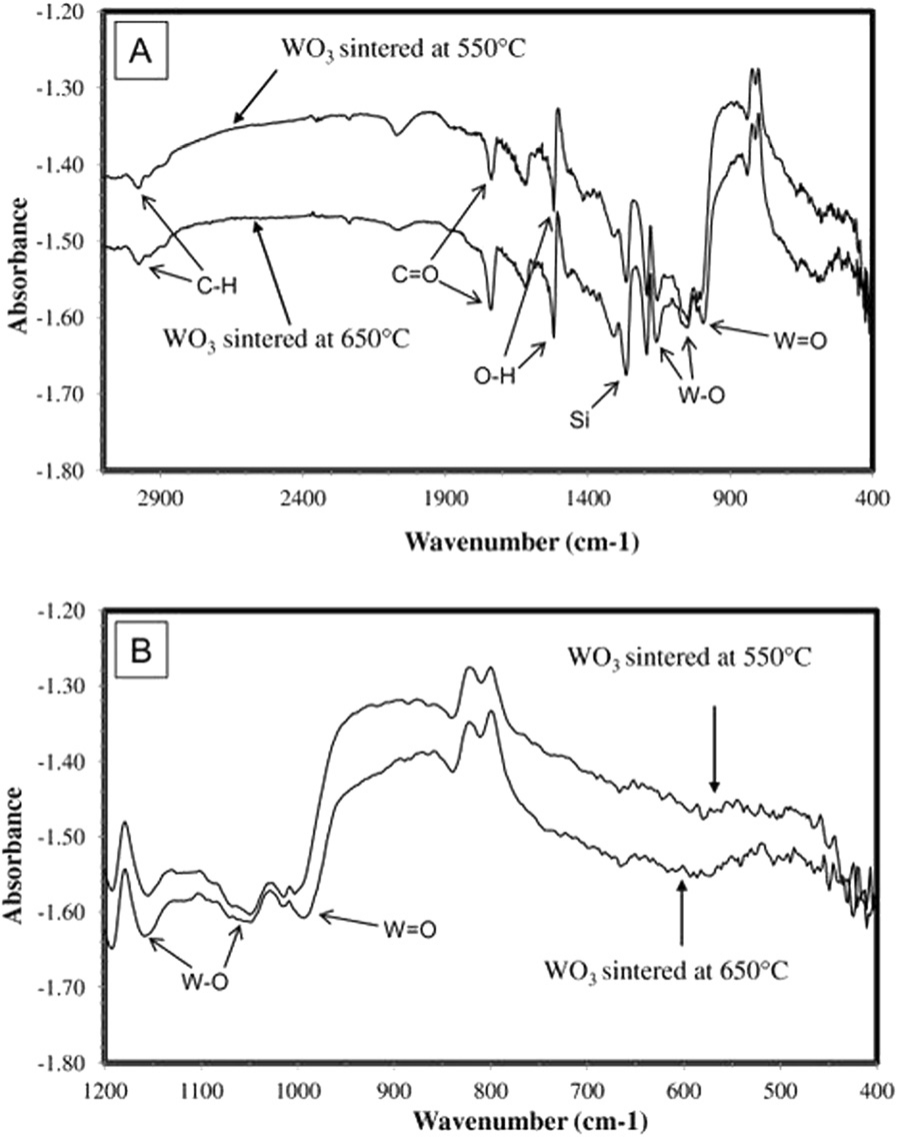
**FTIR measurements for WO**_**3 **_**nanoflakes sintered at 550°C and 650°C. (A)** Total IR spectra. **(B)** Perturbation region within 400 to 1,200 cm^-1^.

*In situ* FTIR spectroscopy of Q2D WO_3_ nanoflakes was utilized to determine surface chemistry and surface reactions of the developed crystalline nanostructures [36]. This is a very powerful technique particularly for elucidating changes in hydration and hydroxylation that occur on the surface of Q2D nanoflakes. The FTIR spectra for Q2D WO_3_ nanoflakes sintered at 550 and 650°C, respectively, are presented in Figure 6. They illustrate the bonding characteristics of the functional groups in the sol-gel prepared and exfoliated Q2D WO_3_. The higher surface area enables the detection of bands owing to surface OH and adsorbed water in the 3,700 to 3,100 cm^-1^ region (not shown in presented Figure 6). Specifically, the sharp peaks at 1,620 cm^-1^ are various O-H stretching modes due to H_2_O bending mode. Generally, about 40% of the total adsorbed water remains strongly bound to the surface up to 150°C [37]. Weak C-H stretching modes at 2,991 cm^-1^ were also observed.

Considering that WO_3_ contains cations in the highest degree of oxidation (+6), CO molecules do not adsorb on its surface because of full coordination. The frequency values obtained in spectra of CO adsorbed on Q2D WO_3_ nanoflakes shifted to the lower values compared to the assignments represented for microstructured WO_3_[38]. This is connected with the fact that in the analysed Q2D WO_3_ nanoflakes, the degree of oxidation on some parts of the WO_3_ surface has been changed and few WO_3-*x*_ sites appeared on the surface of nanoflakes causing CO adsorption. It should be noted that some residual hydrated WO_3_ is most likely present in the sample because hydrated WO_3_ is formed in the sol-gel process and then converted to β-WO_3_ during sintering [37,39]. In the obtained spectra, the peaks in the fingerprint region, namely, at 1,048 and 1,161 cm^-1^, are assigned to stretching mode of W-O, whereas the stretch at 984 cm^-1^ is due to W = O vibrations. The W-O stretching modes are less intense, and changes in the low-frequency modes may indicate some modifications in the tungsten-oxide framework. This is possibly owing to the fact that the surface of exfoliated Q2D WO_3_ itself contains various defects. In general, the majority of experimental phenomena discussed above were associated to adsorption on expected sites of oxide nanoflake surface (co-ordinatively unsaturated cations, hydroxyls and their pair). However, the appearance of the most active surface centres suggests a connection with defects in nanoflakes [38,40]. The other factors influencing properties of the ‘real’ oxide surfaces are (i) the presence of different lattice defects in the surface layer of nanoflake and (ii) their chemical composition, which in many cases, may differ from that in the microstructured material. There was also one stretch observed at 1,265 cm^-1^ (Si), which directly relates to the substrate platform. The WO_3_ FTIR spectra also indicated that there were no impurities present in the prepared and exfoliated samples.

**Figure 7:**
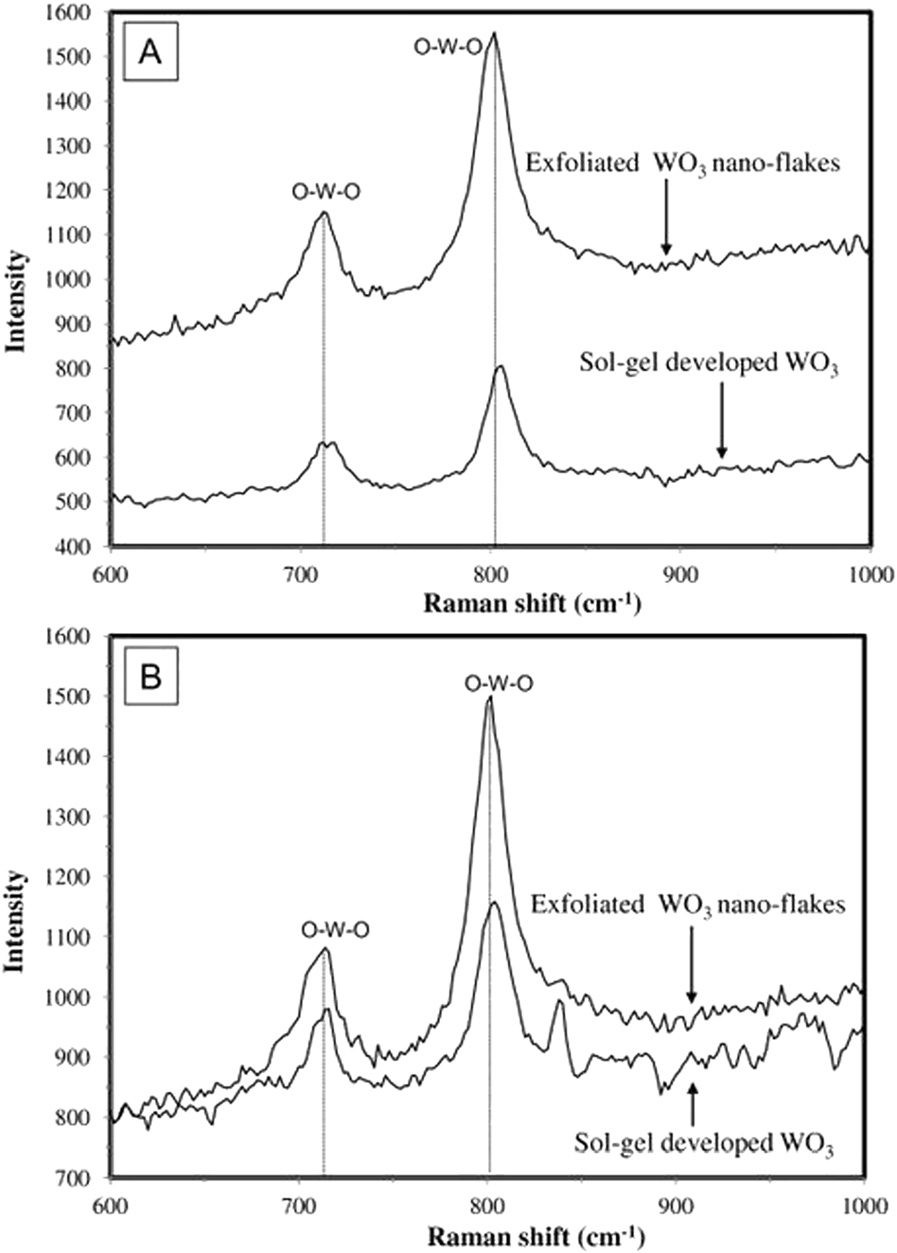
**Raman spectra (perturbation region within 600 to 1,000 cm**^**-1**^**) for sol-gel-developed WO**_**3 **_**and exfoliated Q2D WO**_**3 **_**nanoflakes.** Sintered at 550°C **(A)** and 650°C **(B)**, respectively.

Raman spectroscopy was employed to determine the vibration and rotation information in relation to chemical bonds and symmetry of molecules in sol-gel-developed WO_3_, sintered at 550° and 650°C, respectively, and exfoliated ultra-thin Q2D WO_3_. Raman spectra for sol-gel-developed WO_3_ and exfoliated Q2D WO_3_ nanoflakes in the perturbation area of the spectrum are shown in Figure 7. In both cases, Raman peaks corresponding to WO_3_ were observed. The bending modes of WO_3_ are usually located between 600 and 900 cm^-1^, while the stretching modes can be observed between 200 and 500 cm^-1^[41]. The prominent band situated at 802 cm^-1^ has been assigned to the symmetric stretching mode of terminal (W^6+^ = O) groups which may also be vibrationally coupled [42]. This peak represents lattice discontinuities which lead to short-range (lattice) order. The presence of O-W-O bond is typically associated with β-WO_3_[43]. There were no other substantial peaks noted, suggesting that no impurities were present in the samples. Bridging (O-W-O) vibrations, which occur around 700 cm^-1^, are influenced significantly by hydration [30], and as a result, the recorded 712 cm^-1^ band can be used as a spectral marker for hydration level of WO_3_[44]. However, care should be exercised using this approach, since the crystalline hexagonal phase (h-WO_3_) also exhibits bands at these frequencies but is likely to be absent in sample prepared without a thermal annealing step.

It is noteworthy that the intensity of the peaks for the exfoliated Q2D WO_3_ nanoflakes sintered at 550°C was about two times higher than that the strength of peaks for the same sol-gel-developed WO_3_. At the same time, the magnitude of the peaks for exfoliated Q2D WO_3_ nanoflakes sintered at 650°C was just ~0.3 to 0.5 times higher compared to the intensity of peaks for their sol-gel-developed WO_3_ counterparts. This finding has confirmed the thickness-dependent properties of ultra-thin Q2D WO_3_. Following sintering at 550°C, there is a reduction in the spectral line width consistent with greater crystalline phase formation. Well-defined bands 712 and 802 cm^-1^ modes exhibit significant changes, with the mode at 712 cm^-1^ being particularly sensitive to the cation intercalation [45]. Consequently, these results and observations open up a possibility for the future potential use of 2D WO_3_ as suitable nanomaterial for various sensing platforms [1,10,46] and reaffirmed that the sintering temperature of 550°C more suitable for synthesis of 2D WO_3_ than 650°C aiming their further exfoliation and cation intercalation.

**Figure 8:**
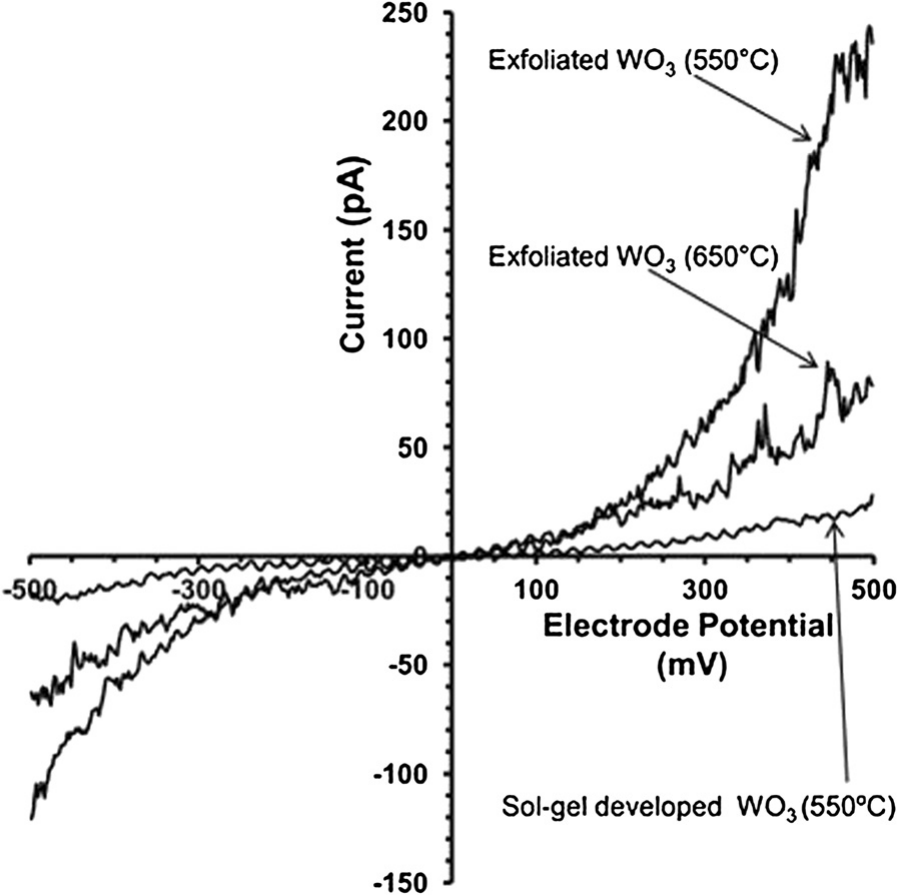
***I-V *curves derived from CSFS-AFM images for sol-gel-developed WO**_**3 **_**and exfoliated Q2D WO**_**3 **_**nanoflakes.** These *I-V* curves have been obtained by averaging the current values recorded independently for different DC sample bias.

Electrical CSFS-AFM measurements revealed and further proved the thickness-dependent properties of ultra-thin Q2D WO_3_*. I-V* curves for the sol-gel-developed WO_3_ nanostructures sintered at 550°C and for exfoliated ultra-thin Q2D WO_3_ nanoflakes sintered at 550°C and 650°C are presented in Figure 8. The current is measured by averaging the data values on the current image corresponding to the same voltage. There were neither significant oxidation nor reduction peaks recorded during scans. Non-linear behaviour for all samples was observed. This behaviour is typical for the semiconductor nature of the WO_3_[21]. However, the electrical performance showed significant difference between the sol-gel-developed WO_3_ nanostructures and exfoliated Q2D WO_3_ nanoflakes. It is clearly exhibited that the measured current for Q2D WO_3_ was about from 5 (650°C) to 10 (550°C) times higher than the measured current for the sol-gel-developed WO_3_ nanostructures. This fact confirms that the CFSF-AFM current originates from the local properties of the material at the tip-sample contact. The higher electrical activity and therefore greater currents for the exfoliated Q2D WO_3_ nanoflakes appeared to be more related to higher heterogeneous electron exchange rate caused by the quantum confinement effects within the few-layers limit [47]. Consequently, Q2D WO_3_ nanoflakes can offer reduced power dissipation because of smaller short channel effects [48]. Furthermore, the electrical measurements have also proved that the sintering temperature of 550°C is more suitable and superior for the development of Q2D WO_3_ nanoflakes with enhanced properties.

**Figure 9:**
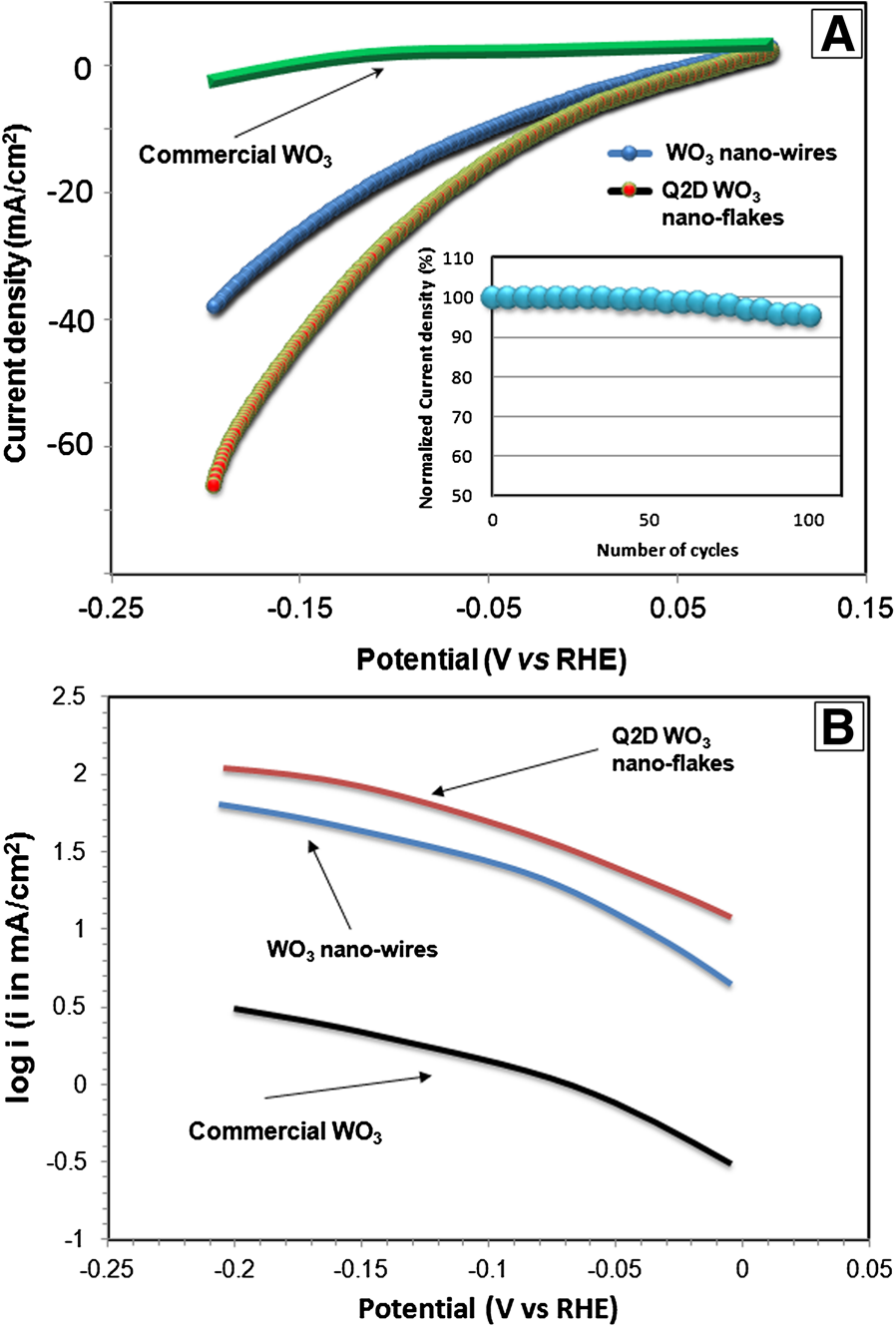
**Linear voltammograms of commercial WO**_**3**_**, Q2D WO**_**3 **_**nanoflakes and hexagonal WO**_**3 **_**nanowires in 1.0 M H**_**2**_**SO**_**4 **_**solution (A).** Insert, measured electrochemical stability for 100 cycles at -0.1 V (*vs* RHE). **(B)** Corresponding Tafel plots obtained from the LSV.

LSV voltammograms for commercial WO_3_ (surface area = 3 m^2^ g^-1^) [49] and Q2D β-WO_3_ nanoflakes sintered at 550°C were recorded in a potential region of +0.1 to -0.2 V at a scan rate of 50 mV s^-1^ in 1.0 M H_2_SO_4_ solution. The results are presented in Figure 9A. Recent LSV results for hexagonal WO_3_ nanowires [15] in the same solution and at the same scan rate and potential range were also provided for comparison. It is clearly shown that the commercial WO_3_ exhibited very low catalytic activity towards electrochemical reaction for HER in this potential region, whereas Q2D β-WO_3_ nanoflakes sintered at 550°C displayed improved electro-catalytic activity. The observed electrochemical stability was recorded for 100 consecutive cycles in the solution (insert in Figure 9A) and confirmed only ~5% decrease from the initial current density. It can therefore be concluded that the activity of electrochemical reaction in this acid media of Q2D WO_3_ nanoflakes remains high after a substantial number of working cycles. In contrast to the commercial WO_3_, which consists of randomly oriented particles of the different size, the developed Q2D β-WO_3_ nanoflakes possess high aspect ratio and high crystallinity which stipulates the high electro-catalytic activity.

The Tafel plots (Figure 9B) were constructed from the LSV voltammograms in the voltage region of -0.02 to -0.20 V. The Tafel slopes for commercial WO_3_, Q2D WO_3_ nanoflakes and hexagonal WO_3_ nanowires are -157, -112 and -116 mV decade^-1^, respectively [15]. The lower Tafel slope obtained from Q2D WO_3_ nanoflakes indicates that it is a superior material as a hydrogen production electrode of HER compared to hexagonal WO_3_ nanowires [15] and commercial WO_3_[49]. This could be attributed to the enhanced electrons transfer kinetics in ultra-thin Q2D nanoflakes, which can play a decisive role as a driving force to reduction of the electrochemical resistance [50]. These results demonstrate that Q2D β-WO_3_ nanoflakes developed via two-step sol-gel-exfoliation method can be effective electrode materials with improved HER activity.

## Conclusions

Orthorhombic Q2D β-WO_3_ nanoflakes, typically with lengths and widths of the order of 50 to 100 nm and thickness of 7 to 9 nm were produced by a two-step sol-gel-exfoliation method. It was experimentally determined that exfoliation of the ultra-thin Q2D β-WO_3_ nanoflakes was only possible at nanostructures sintered at 550 and 650°C. Spectral evidence for β-WO_3_ phase exists in the Raman measurements. This is also consistent with the absence of other crystalline phases in the XRD measurements of this material. CSFS-AFM, FTIR, Raman and electrochemical measurements further confirmed that the annealing temperature of 550°C is the most acceptable sintering temperature for WO_3_, if ultra-thin Q2D β-WO_3_ nanoflakes with thickness of ~7 to 9 nm have to be obtained. The results of the conducted research have reassured the thickness-dependent properties of the ultra-thin Q2D WO_3_ nanoflakes and demonstrated that Q2D WO_3_ nanoflakes can be excellent electro-catalytic material for HER with high activity and stability in water. The present study also illustrates the fundamental role the nanostructure of WO_3_ on the catalytic performance. The high surface-to-volume ratio of Q2D WO_3_ nanoflakes, controllable deposition and compatibility with existing semiconductor fabrication infrastructure suggest that the reported Q2D β-WO_3_ nanostructures can be utilized in new generation of low-cost oxide semiconductor functional devices including solar cells and various sensing platforms. Moreover, both the fabrication process and its framework have great compatibility with other emerging Q2D semiconductors and conductors such as graphene.

## Competing interests

The authors declare no competing interests.

## Authors' contributions

S.Z. conceived the idea, designed the experiments, conducted XRD, EDX and impedance measurements and analysed the data. E.K synthesized Q2D WO_3_ nanoflakes, characterized them with CSFS-AFM, SEM, FTIR, Raman and electrochemical measurements and analysed the data. S.Z. and E.K organized, wrote and edited the paper. All authors contributed to the discussion and preparation of the manuscript. All authors read and approved the final manuscript.

## Authors' information

S.Z. obtained his Ph.D. in Materials Science and Engineering in 1991. He has combined experience as Research Scientist working at the different universities in Australia, Japan and Europe and industrial environments for more than 23 years. He is a Principal Research Scientist at Materials Science and Engineering Division of CSIRO. His research interests lie in the area of the development, design and evaluation of new functional nanomaterials for state-of-the-art functional devices. He is also Chairman of *FP-011-02* Technical Committee of Standards Australia International and a Head of the Australian delegation in International Standards Organization: *ISO TC21/SC8* Technical Committee since 2005. He has published 2 monographs, 6 chapters to books and more than 170 peer-reviewed scientific publications. He is a recipient of the 2007, 2011 and 2013 Australian Academy of Science/Japan Society for Promotion of Science and 2010 Australian Government Endeavour Executive Awards for his work on nanostructured materials.

E.K. was awarded a BSc (Applied Chemistry) from the University of RMIT, Victoria, Australia (1997). From 1998 until 2004, Eugene worked as a Research Project Officer at Scientific Services Laboratory, Melbourne, Australia. During this period, he was responsible for both technical and management components of Sample and Compliance testing of fire equipment, including detection equipment. Eugene has joined CSIRO Materials Science and Engineering Division in 2004. His current research involves development of nanostructured semiconductor materials for various functional devices.
